# Impact of H1N1 on Socially Disadvantaged Populations: Systematic Review

**DOI:** 10.1371/journal.pone.0039437

**Published:** 2012-06-25

**Authors:** Andrea C. Tricco, Erin Lillie, Charlene Soobiah, Laure Perrier, Sharon E. Straus

**Affiliations:** 1 Li Ka Shing Knowledge Institute of St Michael’s Hospital, Toronto, Ontario, Canada; 2 Child Health Evaluative Sciences, The Hospital for Sick Children, Toronto, Ontario, Canada; 3 Continuing Education and Professional Development, University of Toronto, Toronto, Ontario, Canada; 4 Division of Geriatrics, Faculty of Medicine, University of Toronto, Toronto, Ontario, Canada; University of Calgary & ProvLab Alberta, Canada

## Abstract

**Background:**

The burden of H1N1 among socially disadvantaged populations is unclear. We aimed to synthesize hospitalization, severe illness, and mortality data associated with pandemic A/H1N1/2009 among socially disadvantaged populations.

**Methods/Principal Findings:**

Studies were identified through searching MEDLINE, EMBASE, scanning reference lists, and contacting experts. Studies reporting hospitalization, severe illness, and mortality attributable to laboratory-confirmed 2009 H1N1 pandemic among socially disadvantaged populations (e.g., ethnic minorities, low-income or lower-middle-income economy countries [LIC/LMIC]) were included. Two independent reviewers conducted screening, data abstraction, and quality appraisal (Newcastle Ottawa Scale). Random effects meta-analysis was conducted using SAS and Review Manager.

**Conclusions/Significance:**

Sixty-two studies including 44,777 patients were included after screening 787 citations and 164 full-text articles. The prevalence of hospitalization for H1N1 ranged from 17–87% in high-income economy countries (HIC) and 11–45% in LIC/LMIC. Of those hospitalized, the prevalence of intensive care unit (ICU) admission and mortality was 6–76% and 1–25% in HIC; and 30% and 8–15%, in LIC/LMIC, respectively. There were significantly more hospitalizations among ethnic minorities versus non-ethnic minorities in two studies conducted in North America (1,313 patients, OR 2.26 [95% CI: 1.53–3.32]). There were no differences in ICU admissions (n = 8 studies, 15,352 patients, OR 0.84 [0.69–1.02]) or deaths (n = 6 studies, 14,757 patients, OR 0.85 [95% CI: 0.73–1.01]) among hospitalized patients in HIC. Sub-group analysis indicated that the meta-analysis results were not likely affected by confounding. Overall, the prevalence of hospitalization, severe illness, and mortality due to H1N1 was high for ethnic minorities in HIC and individuals from LIC/LMIC. However, our results suggest that there were little differences in the proportion of hospitalization, severe illness, and mortality between ethnic minorities and non-ethnic minorities living in HIC.

## Introduction

In 2009, a novel H1N1 influenza virus strain circulated, which gave rise to the 2009 H1N1 pandemic (influenza A/Mexico/2009 (H1N1)). The H1N1 pandemic was associated with a high burden of illness in terms of hospitalizations, severe illness, absenteeism, and cost. For example, in the United States (US), over 43,677 laboratory-confirmed cases of pandemic H1N1 2009 were reported [1]. Using a mathematical model, it was estimated that between 1.8 million to 5.7 million cases occurred, including 9,000 to 21,000 hospitalizations in the US [1]. In Spain, the average work absenteeism due to the 2009 H1N1 pandemic ranged from 9 to 30.5 days [2]. The economic burden for those with confirmed influenza was estimated as €144,773,577 in Spain (95% confidence interval, CI: 13,753,043-383,467,535). In Italy, the estimated economic burden due to laboratory-confirmed H1N1 2009 pandemic ranged from €1.3 to €2.3 billion [3]. In Australia, the economic burden of treating H1N1-admitted patients to the intensive care unit (ICU) was over AU $65,000,000 [4]. These estimates indicate that a significant burden of illness was observed due to the 2009 H1N1 pandemic.

Previous reviews of the 2009 H1N1 pandemic reported that the majority of cases occurred among young to middle-aged adults often in those without comorbidity, followed by children and adolescents [5], [6]. Individuals with a greater burden of illness included the elderly, obese individuals, pregnant women, or those with comorbidity [5], [6]. In addition, it has been hypothesized that greater burden of illness was associated with poverty and individuals without access or disproportionate access to healthcare [7]. To examine this further, we aimed to synthesize hospitalization, severe illness, and mortality data associated with pandemic A/H1N1/2009 among socially disadvantaged populations, including low socioeconomic status, ethnic minorities, groups without access or disproportionate access to healthcare, and low-income economy countries or lower-middle-income economy countries [LIC/LMIC].

## Methods

A systematic review protocol was used to guide the methods of our review, based on the Preferred Reporting Items for Systematic Reviews and Meta-analysis (PRISMA) Statement [8]. Our research question was: “what is the evidence that the burden of H1N1 was associated with social disadvantage?” At the time of study conduct, a similar systematic review protocol focusing on H1N1 burden among the socially disadvantaged did not exist.

### Search

An experienced librarian (Perrier) developed the search strategy using medical subject headings (MeSH) and text words. The MEDLINE (OVID interface, 2009 to July 25, 2011) and EMBASE (OVID interface, 2009 to July 25, 2011) electronic databases were searched to identify potentially relevant material. The full search strategy for MEDLINE is presented in Appendix S1, which was modified for EMBASE (available upon request). Targeted searching for low-income and lower-middle-income economies was also conducted in PubMed using the terms “H1N1” and “country of interest”. The Eurosurveillance Journal and the Centers for Disease Control and Prevention (CDC) Morbidity and Mortality Weekly Report were hand-searched from January 2009 until August 1, 2011. The reference lists of included studies or relevant reviews [5], [6], [9] were scanned and a list of included studies was circulated to members of the World Health Organization (WHO) Influenza Programme to ensure all potentially relevant studies were identified.

### Study Selection and Characteristics

Studies reporting the burden (prevalence of hospitalization, severe illness, and mortality) of influenza A/Mexico/2009 (H1N1) among socially disadvantaged populations (e.g., ethnic minorities, low socioeconomic status, groups without access or disproportionate access to healthcare, LIC/LMIC) were included. It was determined *a priori* in discussion with the World Health Organization (organization that commissioned this systematic review) that burden would include hospitalization, severe illness, and mortality. H1N1 had to be laboratory-confirmed through polymerase chain reaction, viral culture, or antibody assay [10], as each laboratory test has various advantages and limitations [11]. For example, antibody assay can detect infections missed by other laboratory methods but the diagnosis of influenza is retrospective and it takes weeks to retrieve the results [11]. According to the Center for Disease Control and Prevention (CDC), the preferred method of pandemic H1N1 2009 influenza ascertainment was polymerase chain reaction and viral culture [12]. Rapid influenza diagnostic tests were not recommended by the CDC (and hence, were excluded from this systematic review), as their sensitivity is low [10].

Ethnic minorities were classified as non-predominant races (e.g., non-Caucasians in predominant Caucasian continents, such as Europe and North America), as well as indigenous populations (i.e., first settlers in a particular territory [9]). LIC (gross national income ≤$1,005) and LMIC (gross national income $1,006–$3,975) were categorized according to the World Bank’s classification of countries [13]. We planned to classify low socioeconomic status as high-school education or less or below the particular country’s poverty line, but we did not identify articles relevant to this type of social disadvantage [14]. Inclusion was not limited by study design, publication status or language. Authors of conference proceedings were contacted to obtain the conference presentation or unpublished work. Two reviewers independently screened the titles and abstracts from the literature search and potentially relevant full-text articles for inclusion using the standardized eligibility criteria. Conflicts were resolved by discussion amongst the team.

### Data Abstraction

A draft data abstraction form was developed, pilot-tested, and modified as necessary. Two reviewers abstracted all of the data independently. Conflicts were resolved by discussion amongst the review team. The following data were abstracted: study characteristics (e.g., study design, country of conduct, time period), patient characteristics (e.g., mean age, percent gender, type of social disadvantage examined) and outcomes (number of hospitalizations, severe illness, deaths). Authors were contacted for further information when the data were not clearly reported. In some instances, multiple studies reported H1N1 data from the same source (i.e., companion reports). When this occurred, the report with the most outcomes of interest or largest sample size was included and the other(s) was used for supplementary material only.

### Validity Assessment

All relevant studies were assessed for risk of bias using the Newcastle Ottawa Scale (NOS) [15]. The NOS evaluates nonrandomized studies such as case-control and cohort studies and consists of 3 domains: selection, comparability, and exposure. A full explanation of the NOS can be found in Appendix S2.

### Quantitative Data Synthesis

Random effects meta-analysis [16] was conducted to determine the prevalence of hospitalization, ICU admission, and mortality that occurred in high-income economy countries (HIC) and LIC/LMIC, separately, as well as for the proportion of ethnic minorities and non-ethnic minorities experiencing these outcomes in HIC. Confounding was examined through meta-analyses of the proportion of patients with comorbidity, pregnancy, and obesity for ethnic minorities versus non-ethnic minorities in HIC. Statistical heterogeneity was examined using the I^2^ and χ^2^ statistics [17]. Analyses were conducted in Review Manager Version 5 [18] and SAS (SAS 9.1 software, SAS Institute Inc., Cary, NC, USA).

## Results

### Flow of Included Studies

**Figure 1:**
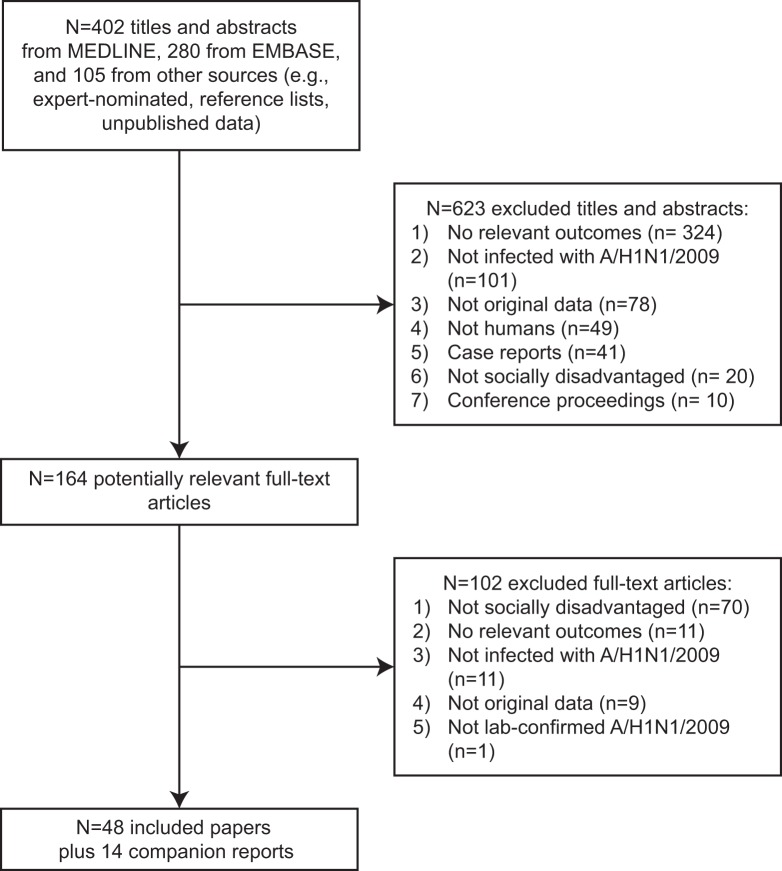
Study flow. This is the flow of citations and articles that were screened for the systematic review.

The literature search identified 787 titles and abstracts; 164 were potentially relevant (Figure 1, Flowchart S1, Checklist S1). From these, 62 articles fulfilled the eligibility criteria [19]–[80]. Of these, fourteen articles were companion reports and only supplementary data were obtained from them [23], [27], [31], [34], [36], [50], [54], [55], [57], [71], [75]–[78]. All of the studies were written in English and unpublished data were obtained for two studies identified by contacting authors of excluded studies [33], [35].

### Study Characteristics

The majority of the included studies were conducted in HIC, including the United States [20], [21], [24], [28]–[30], [32], [35], [37]–[40], [42], [45], [46], [48], [62], Australia or New Zealand [22], [43], [44], [49], [51], [53], [56], [59], [63], Canada [19], [25], [26], [33], [47], and the United Kingdom [41], [52], [58] (Table 1). Twelve studies were conducted in LIC/LMIC, including Guatemala [64], Morocco [65], [66], Pakistan [80], and India [67]–[78]. All of the included studies were cohort studies.

**Table 1:** Study characteristics.

First author, Year	Time period	Region of conduct	Data source(s)	Number of lab-confirmed H1N1 [confirmation type]	Outcome(s) examined
**ETHNIC MINORITIES**					
Oluyomi-Obi 2010 [19]	March 1–August 31 2009	Manitoba, Canada	St. Boniface Hospital & Health Sciences Centre ICU	889 [PCR]	ICU admission
CDC Sept 2009 [20]	April-August 8 2009	US	CDC influenza-associated pediatric mortality reporting system	36 pediatric deaths [RT-PCR]	Mortality
Martin 2010 [21]	April 1–October 31 2009	North Carolina, US	Duke University Medical Center	123 hospitalizations [RT-PCR, viral culture]	Hospitalization
Paine 2010 [22]*(1)	April 1–November 1 2009	New Zealand	EpiSurv	3067 [PCR, viral culture, antibody assay]	Mortality
Baker 2009 [23] (1)	April 25–August 23 2009	New Zealand	Surveillance data from notifiable disease, general practices, laboratories, Healthline, Ministry of Health ICU, population survey (Flutracker)	3109 [PCR, viral culture, antibody assay]	Hospitalization
Wenger 2011 [24]	April 1– December 31 2009	Alaska, US	Alaska ILI Surveillance Network, Medicaid, Indian Health Service Influenza Awareness System	103 hospitalizations [PCR, viral culture]	Hospitalization, mortality
Zarychanski 2010 [25]	April 2–September 5 2009	Manitoba, Canada	Manitoba Health and University of Manitoba Critical Care	894 (location known for 795) [RT-PCR]	Hospitalization, ICU admission
Helferty 2010 [26]*(2)	April 12 2009– April 3 2010	Canada	PHAC	8678 hospitalizations [RT-PCR, viral culture, antibody assay]	Hospitalization, ICU admission, mortality
Campbell 2010 [27] (2)	April 26–September 26 2009	Canada	PHAC	1479 hospitalizations [RT-PCR, viral culture, antibody assay]	Hospitalization, ICU admission, mortality
Siston 2010 [28]	April 14 -August 21 2009	US	CDC (Pregnancy Flu Line)	788 pregnant women [rRT-PCR, antibody assay, rapid test, viral culture]	Hospitalization, ICU admission, mortality
CDC Dec 2009 [29]	April 15–November 13 2009	US	Multidisciplinary workgroup from 12 state health departments	NR [rapid test, antibody assay, rRT-PCR, viral culture]	Mortality
Chitnis 2010 [30]*(3)	April 23–August 15 2009	Wisconsin, US	Wisconsin Division of Public Health	252 hospitalizations [rRT-PCR]	Hospitalization, ICU admission, mortality
Truelove 2011 [31] (3)	April 15 2009–January 2 2010	Wisconsin, US	Wisconsin Division of Public Health	1266 [rRT-PCR]	Hospitalization, mortality
Dee 2011 [32]	April 15 2009–January 26 2010 hospitalization (pediatric deaths: April 15 2009– March 23 2010)	US	Emerging Infections Program (CDC, 10 state and local health departments, academic institutions, medical providers), CDC Influenza-Associated Pediatric Mortality Surveillance System	5793 hospitalizations [rapid test, RT-PCR, viral culture, documented in medical chart]	Hospitalization, mortality
Jung 2011 [33] *(4) unpublished data	April 16 2009–April 12 2010	Canada	Canadian Critical Care Trials Group	565 critically ill [rRT-PCR, viral culture]	ICU admission
Jouvet 2010 [34] (4)	April 16–August 15 2009	Canada	Canadian Critical Care Trials Group	49 pediatric ICU admissions [PCR, viral culture]	Pediatric ICU admission
Louie 2011 [35]*(5) unpublished data	April 1 2009–August 13 2010	California, US	California Department of Public Health	2476 hospitalizations [rRT-PCR]	Hospitalization, ICU admission, mortality
CDC May 2009 [36] (5)	April 20–May 17 2009	California, US	California Department of Public Health	333 hospitalizations [rRT-PCR]	Hospitalization
Lee 2010 [37]	April 24–July 1 2009	New York City, US	New York City Department of Health and Mental Hygiene, New York City Office of Vital Statistics (death certificates)	47 deaths [rRT-PCR]	Mortality
CDC Aug 2009 [38]	April 24–July 25 2009	Illinois, US	Chicago Department of Public Health	1557 [rRT-PCR]	Hospitalization
CDC Jan 2010 [39]	April 25–May 24 2009	New York City, US	New York City Department of Health and Mental Hygiene (all hospitals)	99 hospitalizations [PCR]	Hospitalization
Kwan-Gett 2009 [40]	April 25–August 7 2009	Washington, US	Public Health–Seattle & King County	565 [viral culture, PCR]	Mortality
Nguyen-Van-Tam 2010 [41]	April 27–Sept 30 2009	UK	The Influenza Clinical Information Network (FluCAN)	631 hospitalizations [rRT-PCR]	Hospitalization
Kumar 2010 [42]	April 28–August 31 2009	Wisconsin, US	Children’s Hospital of Wisconsin	81 hospitalizations [rRT-PCR]	Hospitalization, mortality
Harris 2010 [43]	April-August 2009	Queensland, Australia	Townsville Hospital	360 [rPCR]	Hospitalization, ICU admission, mortality
Kelly 2009 [44]	May-October 2009	Australia	Australian Department of Health and Ageing	NR [lab-confirmed]	Hospitalization, ICU admission, mortality
Jain 2009 [45]	May 1–June 9 2009	US	State health departments reports to the CDC	272 hospitalizations [rRT-PCR]	Hospitalization
Creanga 2010 [46]	May 1–June 30 2009	New York, US	New York City Department of Health and Mental Hygiene	136 hospitalizations [rRT-PCR]	Hospitalization
Bettinger 2010 [47]	May 1–August 31 2009	Canada	The Canadian Immunization Monitoring Program, Active (IMPACT) surveillance data	324 hospitalizations [PCR, antibody assay, viral culture]	Hospitalization
Miller 2010 [48]	May 19–June 30 2009	Utah, US	Four ICUs at three academic hospitals in Salt Lake County	47 ICU admissions [PCR]	ICU admission
Flint 2010 [49]*(6)	June 1–August 31 2009	Northern Territory, Australia	Northern Territory CDC, Royal Darwin Hospital	161 acute care [PCR]	Hospitalization, ICU admission
Flint 2009 [50] (6)	May 29– June 18 2009	Northern Territory, Australia	Northern Territory CDC, Royal Darwin Hospital	81 [PCR]	Hospitalization
Cretikos 2009 [51]	May 15– September 4 2009	New South Wales, Australia	NetEpi, laboratory notifications, hospital emergency department and ambulance dispatch surveillance system, death certificate surveillance	5106 [PCR]	Hospitalization, ICU admission, mortality
Scriven 2009 [52]	June 1–July 21 2009	Birmingham, UK	Heartlands Hospital	3000 [PCR]	Hospitalization, severe illness
Webb 2009 [53]*(7)	June 1–August 31 2009	New Zealand, Australia	Australian and New Zealand Intensive Care Research Centre	722 ICU admissions [PCR, serology]	ICU admission
Knight 2010 [54] (7)	June 1–August 31 2009	Australia, New Zealand	Australian and New Zealand Intensive Care Research Centre, Australasian Maternity Outcomes Surveillance System	59 ICU pregnancy admissions [confirmed H1N1]	ICU admission
ANZIC Investigators 2010 [55] (7)	June 1–August 31 2009	New Zealand, Australia	Australian and New Zealand Intensive Care Research Centre, Australasian Maternity Outcomes Surveillance System	64 ICU pregnancy/postpartum admissions [PCR]	ICU admission
Verrall 2010 [56] *(8)	June 8–August 31 2009	Wellington, New Zealand	Wellington Hospital and Hutt Valley Hospitals	229 hospitalizations [rRT-PCR]	Hospitalization
Dee 2010 [57] (8)	June 17–July 20 2009	Wellington, New Zealand	Hutt Valley Hospital	54 hospitalizations [RT-PCR]	Hospitalization
Sachedina 2010 [58]	June 26 2009– March 22 2010	England, UK	Department of Health, Regional Directors of Public Health, Health Protection Agency’s influenza reference centers	70 deaths [lab-confirmed or H1N1 on death certificate]	Mortality
Kelly 2011 [59]	July 1–December 4 2009	Australia	Influenza Complications Alert Network (FluCAN) sentinel hospitals	465 hospitalizations [rRT-PCR]	Hospitalization, ICU admission
Chien 2010 [60]	July 2–August 29 2009	Taiwan, Republic of China	Taiwan CDC	149 severe illness[RT-PCR]	Pneumonia, respiratory failure
Subramony 2010 [61]	July 15–September 28 2009	Singapore	Singapore General Hospital, Tan Tock Seng Hospital, Alexandra Hospital, National University Hospital, KK Women’s and Children’s Hospital and Changi General Hospital, Mount Elizabeth Hospital, Gleneagles Hospital, Mount Alvernia Hospital, East Shore Hospital, Thompson Medical Centre and Raffles Hospital	1348 hospitalizations [RT-PCR]	Hospitalization, severe illness
Satterwhite 2010 [62]	August 1–October 31 2009	Atlanta, Georgia, US	Emory University Hospital, Grady Memorial Hospital, Emory University Hospital Midtown/Crawford Long Hospital, Atlanta Veterans Affairs Medical Center	109 hospitalizations [PCR]	Hospitalization, ICU admission
Bandaranayake 2011 [63]	January 1–Oct 24 2010	New Zealand	Institute of Environmental Science and Research, Healthstat, Healthline, Episurv, National Minimum Data Set, Pandemic Influenza Mortality Review Committee	1758 [nasopharyngeal swabs]	Hospitalization
**LOW-INCOME ECONOMIES OR LOWER-MIDDLE-INCOME ECONOMIES**					
Reyes 2010 [64]	May-December 2009	Guatemala	National Hospital of Cuilapa, Western Regional Hospital, Health Center of Nueva Santa Rosa	239 [rRT-PCR]	Hospitalization, ICU admission, pneumonia, mechanical ventilation, mortality
Louriz 2010 [65]	June-December 2009	Rabat, Morocco	Ibn Sina University Hospital	186 hospitalizations [RT-PCR]	Hospitalization, ICU admission, mechanical ventilation, mortality
Lahlou 2011 [66]	June 12–December 24 2009	Rabat, Morocco	Mohammed V Military Teaching Hospital	240 [rRT-PCR]	Hospitalization, severe illness, mortality
Jagannatha Rao 2011 [67]	August 2009–April 2010	Karnataka, India	Tertiary care hospital	20 hospitalizations [rRT-PCR]	Hospitalization, severe illness, mortality
Chacko 2010 [68]	August-October 2009	Karnataka, India	Manipal Hospital multidisciplinary ICU	66 hospitalizations [RT-PCR]	Hospitalization, ICU admission, mortality
Parakh 2010 [69]	August 2009–January 2010	New Delhi, India	Kalawati Saran Children’s Hospital	25 pediatric hospitalizations [PCR]	Hospitalization, ICU admission, severe illness, mortality
Ramakrishna 2011 [70]*(9)	August 2009–October 2010	Tamil Nadu, India	Christian Medical College Hospital	629 hospitalizations [rRT-PCR]	Hospitalization, ICU admission, severe illness mortality
Pramanick 2011 [71] (9)	August 5 2009–January 2010	Tamil Nadu, India	Christian Medical College Hospital	164 [rRT-PCR]	Hospitalization, ICU admission, mortality
Puvanalingam 2011 [72]	August 9 2009–January 2010	Tamil Nadu, India	Madras Medical College and Government General Hospital records, Institute of Child Health, Egmore	442 [RT-PCR]	Hospitalization, pneumonia, mortality
Sharma 2010 [73]	September 2009 to January 2010	Delhi, India	GTB Hospital	125 hospitalizations [RT-PCR]	Hospitalization, severe illness, mortality
Chudasama 2011 [74]* (10)	September 1, 2009–February 20, 2010	Gujarat, India	Hospitals in Rajkot city of Saurashtra region	274 hospitalizations [rRT-PCR]	Hospitalization, mortality
Chudasama 2010 [75] (10)	September 1, 2009–February 28, 2010	Rajkot, India	Hospital records, Pediatric Department of Civil Hospital & two other pediatric hospitals of Rajkot city	62 pediatric cases [rRT-PCR]	Hospitalization, mortality
Chudasama 2010 [76] (10)	September 1, 2009–February 20, 2010	Rajkot, India	Hospitals in Rajkot city of Saurashtra region	274 hospitalizations [rRT-PCR]	Hospitalization, mortality
Chudasama 2010 [77] (10)	September 1, 2009–February 20, 2010	Rajkot, India	Hospitals in Rajkot city of Saurashtra region	274 hospitalizations [rRT-PCR]	Hospitalization, mortality
Chudasama 2010 [78] (10)	September 1, 2009–January 31, 2010	Rajkot, India	Hospitals in Rajkot city of Saurashtra region	274 hospitalizations [rRT-PCR]	Hospitalization, mortality
Gupta 2011 [79]	November 2009–February 2010	Rajasthan, India	Umaid Hospital for Women and Children	62 pediatric hospitalizations [rPCR]	Hospitalization, ICU admission, mortality
Naseem 2011 [80]	December 1 2009–May 30 2010	Rawalpindi, Pakistan	Department of Pulmonology and Critical Care, Military Hospital	36 hospitalizations [RT-PCR]	Hospitalization, ICU admission, mechanical ventilation, mortality

**Notes:** *major publication, () denotes companion reports.

**Abbreviations:** CDC Centers for Disease Control, ICU intensive care unit, ILI influenza-like illness, NR not reported, PCR polymerase chain reaction, rRT-PCR real-time reverse transcription-PCR, PH public health, PHAC Public Health Agency of Canada, SES socioeconomic status, UK United Kingdom.

Across the studies, the time period of examination ranged from March 1, 2009 to October 24, 2010. Most studies used PCR to confirm H1N1 infection (44/48); one study reported the use of nasopharyngeal swabs (type of lab-confirmation unspecified) [63], and two studies reported that H1N1 was lab-confirmed, without reporting the type of test used [44], [58]. The majority of studies examined ethnic minority status as the type of social disadvantage (36/48), except for 12 studies that examined H1N1 in LIC/LMIC [64]–[70], [72]–[74], [79], [80].

### Patient Characteristics

Some studies examined differences in the number of hospitalizations, severe illness, and deaths between ethnic minorities and non-ethnic minorities, including Caucasian, European descent, and non-Indigenous populations, which were used as the reference group (Table 2). The number of H1N1-infected individuals ranged from 136 to 5,106 in the included studies (Tables 2 and 3). Few studies reported the percentage of female participants; in those that did report this, it ranged from 24% to 100% (Tables 2 and 3).

**Table 2:** Patient characteristics and outcomes for ethnic minorities.

First author, year	Number of H1N1 patients	Number of H1N1 patients by social disadvantage‡	% female	Median age (range) in years	Number with medical conditions by social disadvantage	Number of hospitalizations	Number of severe illness/ICU	Number of deaths
Oluyomi-Obi 2010 [19]	889	Reports ICU admission by ethnicity	100	Mean 22.6 (16–29)	Pregnant: 5 I, 1 NI	NR	ICU 5 I, 1 NI	NR
CDC Sept 2009 [20]	NR	Reports mortality by ethnicity	50	9 (2 months-17)	Obese:2 B Comorbidity: 1 B, 8 H, 2 A, 13 C [CLD, RAD, CP, seizure, ALL, MD, bronchospasm, htn, DS, pneumonia hx, autism]	NR	NR	6 B, 12 H, 3 A, 15 C
Martin 2010 [21]	NR	Reports severe illness by ethnicity	NR	NR (23–51)	Pregnant: 2 B Comorbidity: 5 B, 1 C [asthma, htn, SLE, seizures, COPD, DM, CHD, CHF]	NR	Cardiac dysfunction: B 5, 1 C	NR
Paine 2010 [22]* Baker 2009 [23]	3067	Reports mortality by ethnicity	NR	All ages	NR	NR	NR	7 I, 8 C, 2 O, 2 U
Wenger 2011 [24]	NR	Reports hospitalization by ethnicity	49	17 (1 week-81)	Underlying disease: 23 I, 8 A/PI, 5 B, 24 C, 4 U	39 I, 9 A/PI, 6 B, 42 C, 7 U	NR	2 I, 4 C
Zarychanski 2010 [25]	795	215 I, 373 NI	52	All ages 5.3(SD 18.8)	NR	74 I, 62 NI	ICU 25 I, 17 NI	NR
Helferty 2010 [26]* Campbell 2010 [27]	NR	Reports hospitalization by ethnicity	50	All ages	NR	607 I, 5484 NI	ICU 115 I, 989 NI	30 I, 258 NI
Siston 2010 [28]	788 pregnant women	141 B, 242 H, 42 A/PI, 9 I, 4 MR, 167 C, 183 U	100	25 (14–43)	Pregnant: 788	103 B, 175 H, 28 A/PI, 7 I, 2 MR, 89 C, 105 U	ICU 15 B, 39 H, 9 A/PI, 31 C, 21 U	2 B, 8 H, 4 A/PI, 13 C, 3 U
CDC Dec 2009 [29]	NR	Reports mortality by ethnicity	NR	All ages	DM: 19 I, 92 NI Asthma: 13 I, 54 NI DM/asthma: 2 I, 238 NI	NR	NR	42 (3.7/100,000) I, 384 (0.9/100,000) NI
Chitnis 2010 [30]* Truelove 2011 [31]	NR	Reports hospitalization by ethnicity	57	28 (0.03–85)	Pregnant: 9 B, 2 H, 1 A, 3 C NMO: 22 B, 11 H, 4 A, 21 C MO: 21 B, 2 H, 6 C Comorbidity: CLD: 8 B, 4 H, 4 A, 13 C; DM: 15 B, 11 H, 4 A, 13 C; Cancer: 4 B, 4 H, 9 C; Hematologic: 14 B, 1 H, 1 A, 1 C	120 (36/100,000) B, 19 (17/100,000) A, 39 H (14/100,000), 68 (1/100,000) C	ICU 19 B, 11 H, 6 A, 23 C	4 B, 2 H, 1 A, 4 C
Dee 2011 [32]	NR	Reports hospitalization by ethnicity	NR	Hospitalizations (all ages), deaths (<18)	NR	1569 (S/S 11/100,000, F/W 30/100,000) B, 1184 (S/S 8/100,000, F/W 31/100,000) H, 329 (S/S 8/100,000, F/W 13/100,000) A/PI, 53 (S/S 4/100,000, F/W 33/100,000) I, 2658 (S/S 3/100,000, F/W 16/100,000) C	NR	45 B, 87 H, 14 A/PI, 6 I, 126 C (pediatric deaths only)
Jung 2011 [33]*† unpublished data; Jouvet 2010 [34]	NR	Reports critically ill by ethnicity	NR	NR	NR	NR	66 I, 499 NI critically ill patients	NR
Louie 2011 [35] * unpublished data; CDC May 2009 [36]	NR	Reports hospitalization by ethnicity	NR	All ages	NR	1024 H, 249 A/PI, 212 B, 16 I, 776 C, 126 U, 73 O	ICU 716 H, 156 A/PI, 132 B, 12 I, 587 C, 99 U, 53 O	238 H, 42 A/PI, 38 B, 4 I, 192 C, 18 U, 7 O
Lee 2010 [37]	NR	Reports mortality by ethnicity	53	43 (7 weeks-82)	NR	NR	NR	17 B, 14 H, 5 A, 9 C, 2 U
CDC Aug 2009 [38]	1557	308 B, 271 H, 47 A/PI, 99 C, 832 U	50	All ages	NR	93 B, 64 H, 10 A/PI, 17 C, 21 U	NR	NR
CDC Jan 2010 [39]	NR	Reports hospitalization by ethnicity	45	All ages	NR	26 B, 38 H, 5 A, 12 C, 18 U	NR	NR
Kwan-Gett 2010 [40]	565	86 B, 127 A, 5 I, 41 other	47	All ages	Comorbidity: 1 A CLD, 1 H chemotherapy, 1 H DM MO: 1 H	NR	NR	1 A, 2 H
Nguyen-Van-Tam 2010 [41]	NR	Reports hospitalization by ethnicity	51	23 (0.25–90)	NR	7 MR, 169 A, 100 B, 59 A/U, 202 C	NR	NR
Kumar 2010 [42]	NR	Reports hospitalization by ethnicity	44	5 (0.1–19.2)	NR	42 B, 17 H, 2 A, 13 C	NR	1 B, 1 H
Harris 2010 [43]	360	95 I, 86 NI	52.5	21 (0.2–90)	Pregnant: 11 I, 80 NI Comorbidity: 71 I, 46 NI DM: 11 I, 3 NI	23 (149/100,000) I, 38 (19/100,000) NI	ICU 2 (13/100,000) I, 7 (4/100,000) NI	1 (7/100,000) I, 4 (2/100,000) NI
Kelly 2009 [44]	NR	Reports hospitalization by ethnicity	NR	NR	NR	803 (150/100,000) I, 4030 NI	ICU 100 (19/100,000) I, 550 NI	24 (5/100,000) I, 162 NI
Jain 2009 [45]	272	Reports hospitalization by ethnicity	49	21 (0.06–86)	NR	83 H, 53 B, 15 I/A/PI, 9 I, 73 C, 2 MR, 37 U	NR	NR
Creanga 2010 [46]	136	Reports hospitalization by ethnicity	100	NR (14–41)	Pregnant: 14 B, 30 H, 2 A, 9 C, 7 U	38 B, 61 H, 6 A, 15 C, 16 U	NR	NR
Bettinger 2010 [47]	NR	Reports hospitalization by ethnicity	45	4.8(0–16)	NR	10 I	NR	NR
Miller 2010 [48]	NR	Reports ICU admission by ethnicity	57	34 (15–62)	NR	NR	ICU 11 H, 1 A, 12 I, 1 B, 22 C	NR
Flint 2010 [49]* Flint 2009 [50]	NR	116 I, 45 NI acute care	52	39 (IQR 15.5–49) I 40 (IQR 19–15) overall	Pregnant: 8 I, 3 NI Obese: 8 I, 6 NI Comorbidity: Asthma: 16 I, 10 NI; COPD: 16 I, 9 NI; Bronchiectasis: 7 I, 2 NI; cardiac disease: 21 I, 8 NI, DM: 22 I, 8 I, Chronic liver disease: 10 I, 2 NI; CKD: 18 I, 2 NI; ND: 5 I, 4 NI; Immunosuppression: 3 I, 3 NI Smoker: 38 I, 5 NI Alcohol: 24 I, 3 NI	92 (296/100,000) I, 39 (29/100,000) NI	36/100,000 I, 7/100,000 NI	NR
Cretikos 2009 [51]	5106	Reports hospitalization by ethnicity	47.8	All ages	NR	96 I, 1118 NI	ICU 14 I, 211 NI	5 I, 43 NI
Scriven 2009 [52]	3000	Reports hospitalization by ethnicity	69	Adults 30.5 (NR)	NR	35 A, 3 B, 14 C	Severe illness: 15 A, 3 B, 1 C	NR
Webb 2009 [53] *; Knight 2010 [54]; ANZIC Investigators 2010 [55]	NR	Reports ICU admission by ethnicity	52.1	40 (IQR: 26–54)	NR	NR	ICU 129 I, 29 A, 483 C, 42 U	NR
Verrall 2010 [56]*; Dee 2010 [57]	NR	Reports hospitalization by ethnicity	NR	Mean 26 (range:0–82)	Chronic lung conditions: 47 I, 45 C/U	134 I (PI 180/100,000; Maori 128/100,000), 74 NI, 21 U (C/U 26/100,000)	NR	NR
Sachedina 2010 [58]	NR	Reports mortality by ethnicity	56	7 (0.25–17)	States “pre-existing health status did not differ between ethnic groups”	NR	NR	27 A, 4 B, 37 C, 2 U
Kelly 2011 [59]	NR	Reports hospitalization by ethnicity	53.8	46 (IQR: 29–58) hospitalizations	NR	67 I, 386 NI	ICU 13 I, 86 NI	NR
Chien 2010 [60]	NR	Reports severe illness by ethnicity	37	RF: 33 (5–73) NRF: 13 (0–59)	NR	NR	2 RF, 3 NRF	NR
Subramony 2010 [61]	NR	Reports hospitalization by ethnicity	51	25 (IQR, 12–50)	NR	599 Chinese [age-gender-adjusted hospitalization rate: 22.1/100,000], 390 Malay [76.3/100,000], 175 ID [53.2/100,000], 131 F [unadjusted: 10.4/100,000], 53 O [unadjusted: 44.2/100,000]	ICU 33 Chinese, 24 Malay, 9 ID, 6 F, 2 O	10 Chinese, 2 Malay, 3 ID, 3 F, 3 U
Satterwhite 2010 [62]	NR	Reports hospitalization by ethnicity	60	41.1 (95% CI: 38.0–44.2)	NR	74 B, 28 C, 7 O/U	ICU admission: 15 B, 6 C, 2 O/U	NR
Bandaranayake 2011 [63]	1758	51/100,000 I, 49/100,000 C, 25/100,000 U	NR	NR	NR	33/100,000 I, 18/100,000 C, 26/100,000 other	NR	NR

**Notes:** *major publication, ‡non-Indigenous and Caucasian are the reference groups unless it was a study conducted in another region (e.g., Chinese considered the reference group in China), Indigenous includes Aboriginals, First Nations, American Indians, Alaskan Natives, Maori, Native Hawaiians, Pacific Islanders, and Torres Strait Islanders, Blacks include African American, African, and Afro-Caribbean, † we obtained unpublished data from the authors of this study in the form of a poster presentation, Π we obtained unpublished data for the state of California from the authors.

**Abbreviations:** A Asian, A/PI Asian/Pacific Islander, ALL acute lymphoblastic leukemia, B Black, C Caucasian, CHD coronary heart disease, CHF congestive heart failure, CI confidence interval, CLD chronic lung disease, COPD chronic obstructive pulmonary disease, CP cerebral palsy, DM diabetes mellitus, DS down syndrome, F foreigners, unspecified,F/W Fall/Winter, H Hispanic, htn hypertension, I Indigenous, ICU intensive care unit, ID Indian descent, MD muscular dystrophy, MO morbid obesity, ND neurological disease, NI non-Indigenous, NMO non-morbid obesity, NRF non-respiratory failure, RAD reactive airway disease, RF respiratory failure, SD standard deviation, SLE systemic lupus erthematosus, S/S Spring/Summer, U unspecified.

**Table 3:** Patient characteristics and outcomes for low-income and lower-middle-income economies.

First author, year	#H1N1 patients	#H1N1 patients by social disadvantage	% female	Median age (range) in years	# medical history by social disadvantage	# hospitalizations	# severe illness/ICU	# deaths
Reyes 2010 [64]	239	239 Guatemalan	40.1	8.8 (0.05–82)	Comorbidity: 53 other respiratory virus, 21 htn, 12 asthma, 2 DM Pregnant: 49	76 Guatemalan	ICU 21 Gautemalan Pneumonia 76 Guatemalan Mechanical ventilation 8 Guatemalan	11 Guatemalan
Louriz 2010 [65]	NR	Reports hospitalization by low-income/lower-middle income economy	43	17.6 (0.08–57; SD 14.8)	Comorbidity: 48 asthma, 13 heart disease, 10 hematologic disease, 4 chronic nephritis, 2 DM Pregnant: 15 Obese 2	186 Moroccan	ICU 20 Moroccan Mechanical ventilation 10 Moroccan	7 Moroccan
Lahlou 2011 [66]	240	240 Morocco	38	All ages 23 (SD 14)	Hospitalization only: Comorbidity: 7 asthma, 5 DM Pregnant: 13 Obese: 2	27 Moroccan	0 Moroccan	0 Moroccan
Jagannatha Rao 2011 [67]	NR	Reports hospitalization by low-income/lower-middle income economy	70	NR (11–80)	Comorobidity: 6 htn	20 ID	Acute respiratory distress syndrome 6 ID, bronchopneumonia 6 ID	5 ID
Chacko 2010 [68]	NR	Reports hospitalization by low-income/lower-middle income economy	42	All ages 35 (IQR: 28.2–42.8) ICU admissions	Comorbidity: 4 htn, 3 DM, 2 asthma, 1 renal failure, 1 immunosuppression, 1 COPD, Obese: 9 Pregnant: 3	66 ID	ICU 38 ID	6 ID
Parakh 2010 [69]	NR	Reports hospitalization by low-income/lower-middle income economy	60	2.5 (0.25–10)	Comorbidity: 16 malnutrition, 1 TB, 1 heart disease	25 ID	Pediatric ICU 7, mechanical ventilation 4	3 ID
Ramakrishna 2011 [70]*; Pramanick 2011 [71]	NR	Reports hospitalization by low-income/lower-middle income economy	??ICU: 68% (wave 1), 51% (wave 2)	ICU: Mean 34.1 (SD 13) wave 1, 36.2 (SD 4.6) wave 2	ICU: Comorbidity: 7 CVD, 8 immunosupressants, 3 DM, 2 respiratory disease MO: 2 Pregnant/postpartum: 15	629 ID	ICU 76 ID Mechanical ventilation 72 ID	48 ID
Puvanalingam 2011 [72]	442	442 ID	44	All ages	Comorbidity: 71 asthma, 39 TB, 32 HTN, 28 DM Alcoholism: 46 Smoking: 35	199 ID (12 pregnant women)	9 ID pregnant women pneumonia	3 ID pregnant women
Sharma 2010 [73]	NR	Reports hospitalization by low-income/lower-middle income economy	Deaths: 50	Deaths: mean 38 (10–65, SD 15.3)	Deaths: Comorbidity: 3 COPD, 3 asthma, 3 asthma, 6 htn, 4 DM, 1 CAD Pregnant: 2 Obese: 3 Smoking: 1 Alcohol: 1	125 ID	Deaths: 12 acute respiratory distress syndrome ID	16 ID
Chudasama 2011 [74]*; Chudasama 2010 [75]; Chudasama 2010 [76]; Chudasama 2010 [77]; Chudasama 2010 [78]	NR	Reports hospitalization by low-income/lower-middle income economy	48.5	27 (0.38–68)	Comorbidity: 27 DM, 24 htn, 15 CLD, 13 CHD, 7 seizures, 2 chronic renal failure Pregnancy: 15	274 ID	NR	71 ID
Gupta 2011 [79]	NR	Reports hospitalization by low-income/lower-middle income economy	24	Mean 5.7 (0.5–18, SD 4.1)	Comorbidity: 30 anemia, 30 malnourished	62 ID	ICU 19 ID	4 ID
Naseem 2011 [80]	NR	Reports hospitalization by low-income/lower-middle income economy	61.1	Adults only Mean 34.24 (SD 13.92)	Hospitalization only: Comorbidity: 2 COPD, 2 DM, 2 CHF Obese: 7 Pregnant: 3 Smoking: 9	36 Pakistani	ICU 10 Pakistani Mechanical ventilation 10 Pakistani	6 Pakistani

**Abbreviations:** CHD coronary heart disease, CHF congestive heart failure, CLD chronic lung disease, COPD chronic obstructive pulmonary disease, DM diabetes mellitus, htn hypertension, ID Indian descent, NR not reported, SD standard deviation.

Two studies included only pregnant women infected with H1N1 [19], [28] and 14 others reported on pregnancies among socially disadvantaged populations [21], [30], [43], [46], [49], [64]–[66], [68], [70], [72]–[74], [80]. No significant differences were observed between the proportion of pregnancies in ethnic minorities and non-ethnic minorities in HIC (n = 765 patients, OR 0.31, 95% CI: 0.03–3.64) [30], [43], [46], [49]. Six studies included only H1N1-infected children and adolescents [20], [42], [47], [58], [69], [79] and two studies reported deaths in pediatric patients (although the number of hospitalizations was for the entire population including all ages for one study) [20], [32]. Twenty-one studies reported comorbidities among the socially disadvantaged; including asthma, chronic lung conditions, heart conditions, and diabetes [20], [21], [24], [29], [30], [40], [43], [49], [56], [64]–[70], [72]–[74], [79], [80]; all studies conducted in LIC/LMIC reported this information. For HIC studies, there was no significant difference observed between ethnic minorities and non-ethnic minorities in terms of prevalent comorbidities (n = 1,203 patients, OR 1.14, 95% CI: 0.63–2.06; Appendix S3) [20], [24], [29], [30], [43], [49]. Ten studies reported on obesity [20], [30], [40], [49], [65], [66], [68], [70], [73], [80] and no significant differences were observed with respect to the prevalence of obesity between ethnic minorities and non-ethnic minorities in HIC studies (n = 500 patients, OR 0.76, 95% CI: 0.46–1.26) [20], [30], [49]. Four studies reported the proportion of patients who were pregnant and no differences were observed in the odds of being pregnant between ethnic minorities and non-ethnic minorities (n = 765 patients, OR 0.31, 95% CI: 0.03–3.64) [30], [43], [46], [49]. Four studies reported on smoking and alcohol use [49], [72], [73], [80]; meta-analysis was not possible because only one study reported on ethnic minorities in a HIC and the other three were conducted in LIC/LMIC.

### Methodological Quality

The majority of the studies used a sample truly representative of the average individual infected with H1N1 in the community (e.g., population-based sample of an entire province or state) or somewhat representative sample of the average individual infected with H1N1 in the community (e.g., pregnant woman from an entire state or province). Ten studies did not use a representative sample; one included volunteers [45], one included only those with severe illness [33], four included individuals with severe illness who died [20], [29], [37], [58], two included pediatric cases in India [69], [79], and two obtained data from one local hospital [43], [49] (Table 4). All of the studies selected the non-exposed cohort from the same community as the exposed cohort and used a structured interview (i.e., self-report) to assess exposure (e.g., social disadvantage), except for studies conducted in LIC/LMIC, for which the data were obtained from hospital records. All of the studies ensured that the patients were not severely ill, hospitalized or dead at the start of the study, except for four studies [20], [29], [37], [58]. All studies assessed the outcomes using record linkage, and the duration of follow-up was deemed appropriate in all of the studies. Most studies did not control for important factors, such as comorbidity (13 studies controlled for comorbidities [22], [29], [32], [33], [41], [43], [48], [49], [56], [58], [60], [61], [63]) and 18 studies had greater than 10% of the patients lost to follow-up or did not describe the follow-up rate [21], [22], [29], [45], [48], [56], [58], [60], [62], [63], [65], [67]–[69], [72], [73], [79], [80].

**Table 4:** Methodological quality.

Study	Representative exposed cohort	Selection of non- exposed cohort	Ascertainment of exposure	Outcome of interest not present at study start	Comparability of cohorts	Assessment of outcome	Long enough follow-up for outcomes to occur	Loss to follow-up
Oluyomi-Obi 2010 [19]	b	a	b	a	c	b	a	a
CDC Sept 2009 [20]	c	a	b	b	c	b	a	a
Martin 2010 [21]	a	a	b	a	c	b	a	d
Paine 2010 [22]	a	a	b	a	a	b	a	d
Wenger 2011 [24]	b	a	b	a	c	b	a	a
Zarychanski 2010 [25]	a	a	b	a	c	b	a	c
Helferty 2010 [26]	a	a	b	a	c	b	a	c
Siston 2010 [28]	b	a	b	a	c	b	a	a
CDC Dec 2009 [29]	c	a	b	c	a	b	a	d
Chitnis 2010 [30]	a	a	b	a	c	b	a	a
Dee 2011 [32]	a	a	b	a	a	b	a	c
Jung 2011 [33] unpub.	c	a	b	a	a	b	a	a
Louie 2011 [35] unpub.	a	a	b	a	c	b	a	a
Lee 2010 [37]	c	a	b	a	c	b	a	a
CDC Aug 2009 [38]	a	a	b	a	c	b	a	a
CDC Jan 2010 [39]	a	a	b	a	c	b	a	a
Kwan-Gett 2010 [40]	a	a	b	a	c	b	a	a
Nguyen-Van-Tam 2010 [41]	a	a	b	a	a	b	a	b
Kumar 2010 [42]	b	a	b	a	c	b	a	a
Harris 2010 [43]	c	a	b	a	a	b	a	c
Kelly 2009 [44]	a	a	b	a	c	b	a	a
Jain 2009 [45]	c	a	b	b	c	b	a	d
Creanga 2010 [46]	b	a	b	a	c	b	a	a
Bettinger 2010 [47]	b	a	b	a	c	b	a	c
Miller 2010 [48]	a	a	b	a	a	b	a	d
Flint 2010 [49]	c	a	b	a	a	b	a	a
Cretikos 2009 [51]	a	a	b	a	c	b	a	a
Scriven 2009 [52]	a	a	b	a	c	b	a	a
Webb 2009 [53]	a	a	b	a	c	b	a	b
Verrall 2010 [56]	b	a	b	a	a	b	a	d
Sachedina 2010 [58]	c	a	b	b	a	b	a	d
Kelly 2011 [59]	a	a	b	a	c	b	a	b
Chien 2010 [60]	b	a	b	a	a	b	a	d
Subramony 2010 [61]	a	a	a	a	a	b	a	b
Satterwhite 2010 [62]	a	a	a	a	c	b	a	d
Bandaranayake 2011 [63]	a	a	a	a	a	b	a	d
Reyes 2010 [64]	b	a	a	a	c	b	a	b
Louriz 2010 [65]	b	a	a	a	c	b	a	d
Lahlou 2011 [66]	b	a	a	a	c	b	a	a
Jagannatha Rao 2011 [67]	b	a	a	a	c	b	a	d
Chacko 2010 [68]	b	a	a	a	c	b	a	d
Parakh 2010 [69]	c	a	a	a	c	b	a	d
Ramakrishna 2011 [70]	b	a	a	a	c	b	a	a
Puvanalingam 2011 [72]	b	a	a	a	c	b	a	d
Sharma 2010 [73]	b	a	a	a	c	b	a	d
Chudasama 2011 [74]	b	a	a	a	c	b	a	a
Gupta 2011 [79]	c	a	a	a	c	b	a	d
Naseem 2011 [80]	b	a	a	a	c	b	a	d

**Note:** Please see Appendix S2 for an explanation of each methodological quality component. Briefly, each item was appraised as follows:

1) Representativeness: a) truly representative, b) somewhat representative, c) selected group of users, d) no description of the derivation of the cohort.

2) Selection: a) drawn from the same community as exposed cohort, b) drawn from a different source, c) no description.

3) Ascertainment: a) secure record, b) structured interview, c) written self report, d) no description.

4) Demonstration: a) yes, b) no.

5) Comparability: a) study controls for age or gender, b) study controls for any additional factor, c) no control.

6) Assessment: a) independent blind assessment, b) record linkage, c) self report, d) no description.

7) Follow-up: a) yes, b) no.

8) Adequacy: a) complete follow up, b) subjects lost to follow up unlikely to introduce bias, c) large loss to follow-up, d) no statement.

### Hospitalization

**Figure 2:**
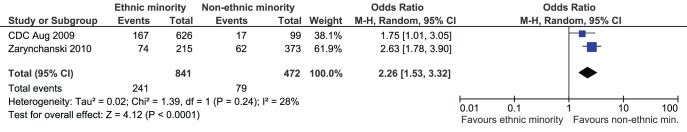
Meta-analysis of hospitalizations among ethnic minorities versus non-ethnic minorities in North America. Favours ethnic minority means that ethnic minorities experienced a lower proportion of H1N1 hospitalizations compared to non-ethnic minorities. Favours non-ethnic minorities means that non-ethnic minorities experienced a lower proportion of H1N1 hospitalizations compared to ethnic minorities.

Twenty-four studies reported hospitalization data broken down by ethnicity (Table 2) [24]–[26], [28], [30], [32], [35], [38], [39], [41]–[47], [49], [51], [52], [56], [59], [61]–[63]. Four studies reported the number of H1N1-infected individuals by ethnicity and the prevalence hospitalization among those with H1N1 ranged from 24–27% among ethnic minorities and 17–87% among non-ethnic minorities in HIC [25], [38], [43], [49]. Two studies were not included in the meta-analysis due to concerns about confounding and inclusion of a non-representative sample [43], [49]. Once these studies were excluded, there was a significantly greater proportion of ethnic minority versus non-ethnic minority hospitalizations (n = 1,313 patients, OR 2.26, 95% CI: 1.53–3.32, I^2^ = 28%; Figure 2) [25], [36]. Similarly, a study including pregnant women infected with H1N1 reported a significantly higher proportion of hospitalizations among ethnic minorities (72%) versus non-ethnic minorities (53%; OR based on this study: 2.27, 95% CI: 1.57–3.28) [28].

All of the studies conducted in LIC/LMIC reported hospitalization data for individuals with H1N1 (Table 3). The prevalence of hospitalization among those infected with H1N1 was 32% in Guatemala [64], 11% in Morocco [66], and 45% in India [72]. Prevalence meta-analysis was not possible because only one study was conducted in each country.

### Severe Illness

Nineteen of the included studies reported the number of H1N1 patients experiencing severe illness broken down by ethnicity, including intensive care unit (ICU) admission [19], [21], [25], [26], [28], [30], [33], [35], [43], [44], [48], [49], [51]–[53], [59], [62], severe illness unspecified [61], and pneumonia or respiratory failure [60]. One study of H1N1-infected pregnant women observed similar proportions of ICU admissions between ethnic minorities (15%) and non-ethnic minorities (19%; OR based on this study: 0.74, 95% CI: 0.46–1.20) [28]. One study observed a similar relationship for patients admitted to the ICU among all H1N1-infected patients in Australia (OR 0.24, 95% CI: 0.05–1.20) [43], while another study observed a greater proportion of ICU admissions among ethnic minorities in Manitoba (OR 2.76, 95% CI: 1.45–5.23) [25]. Meta-analysis was not conducted because the Australian study was affected by confounding and did not use a representative sample [43]. In LIC/LMIC, the proportion of ICU admissions among H1N1-infected was 9% in Guatemala [64] and 0% in Morocco [66].

**Figure 3:**
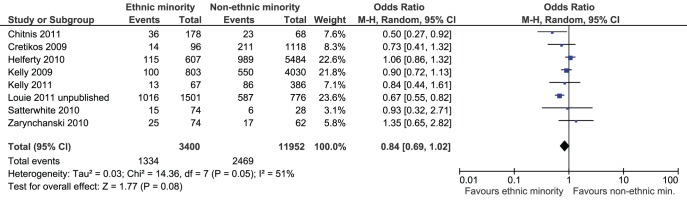
Meta-analysis of ICU admissions among ethnic minorities versus non-ethnic minorities. Favours ethnic minority means that ethnic minorities experienced a lower proportion of intensive care unit (ICU) admissions due to H1N1 compared to non-ethnic minorities. Favours non-ethnic minorities means that non-ethnic minorities experienced a lower proportion of ICU admissions due to H1N1 compared to ethnic minorities.

One study of H1N1-infected pregnant women observed a significantly greater proportion of ICU admissions among non-ethnic minority hospitalizations (35%) versus ethnic minority hospitalizations (35%; OR based on this study: 0.47, 95% CI: 0.28–0.79) [28]. Eight studies reported ethnicity data on the proportion of patients admitted to the ICU among those hospitalized in HIC and were meta-analyzed [25], [26], [30], [35], [44], [51], [59], [62]. The prevalence ranged from 6–68% among ethnic minorities and 7–76% among non-ethnic minorities. One study was not included in the meta-analysis due to concerns about confounding and inclusion of a non-representative sample [43]. Excluding this study, there were no differences in ICU admissions among hospitalized non-ethnic minorities compared to ethnic minorities (n = 15,352 patients, OR 0.84, 95% CI: 0.69, 1.02, I^2^ = 51%, Figure 3).

All of the studies conducted in LIC/LMIC reported data on severe illness (Table 3). Two studies conducted in India reported ICU admission among hospitalized adults and the pooled prevalence was 34% (95% CI: 0–79%) [68], [70]. Two studies reported this information among Indian children and the pooled prevalence was 30% (95% CI: 20–40%) [69], [79].

### Mortality

**Figure 4:**
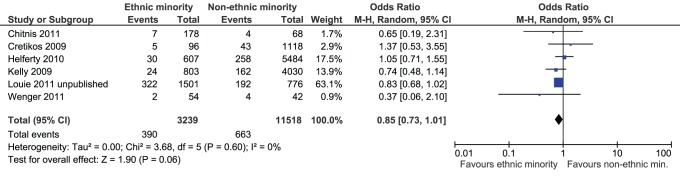
Meta-analysis of mortality among hospitalized ethnic minorities versus hospitalized non-ethnic minorities. Favours ethnic minority means that ethnic minorities experienced a lower proportion of deaths due to H1N1 among hospitalized patients compared to non-ethnic minorities. Favours non-ethnic minorities means that non-ethnic minorities experienced a lower proportion of deaths due to H1N1 among hospitalized patients compared to ethnic minorities.

Fifteen studies reported the number of deaths by ethnicity [20], [22], [24], [26], [29], [30], [32], [33], [35], [37], [40], [43], [44], [51], [58]. One study of pregnant women infected with H1N1 observed a significantly lower proportion of deaths among H1N1-infected pregnant ethnic minorities (3%) versus pregnant non-ethnic minorities (15%; OR based on this study: 0.19, 95% CI: 0.09–0.43) [28]. None of the others reported the proportion of deaths among H1N1-infected individuals. This study also observed a significantly lower death rate among pregnant ethnic minority hospitalizations (4%) versus pregnant non-ethnic minority hospitalizations (15%; OR 0.27, 95% CI: 0.12–0.61) [28]. Mortality ranged from 1–21% among ethnic minorities and 4–25% among non-ethnic minorities in HIC. Six studies reported the proportion of deaths among hospitalized patients and were meta-analyzed [24], [26], [30], [35], [44], [51]. One study was not included in the meta-analysis due to confounding and inclusion of a non-representative sample [43]. Excluding this study, there were no differences in the proportion of deaths among hospitalized non-ethnic minorities compared to ethnic minorities (n = 14,757 patients, OR 0.85, 95% CI: 0.73–1.01, I^2^ = 0%, Figure 4).

All of the studies conducted in LIC/LMIC reported mortality (Table 3). Five studies conducted in India reported the number of deaths among hospitalized adults and the pooled prevalence was 15% (95% CI: 7–23%) [67], [68], [70], [73], [74]. Two reported this information among Indian children and the pooled prevalence was 8% (95% CI: 2–13%) [69], [79].

## Discussion

We conducted a systematic review on the occurrence of hospitalization, severe illness, and mortality among socially disadvantaged groups of the population. The prevalence of hospitalization among those with H1N1 ranged from 24–27% among ethnic minorities and 17–87% among non-ethnic minorities in HIC and was 11–45% in LIC/LMIC. The proportion of patients admitted to the ICU among those with H1N1 ranged from 2–12% among ethnic minorities and 5–8% among non-ethnic minorities in HIC and was 0–9% in LIC/LMIC. The proportion of ICU admissions among hospitalized patients ranged from 6–68% among ethnic minorities and 7–76% among non-ethnic minorities in HIC. The pooled prevalence of ICU admission among hospitalizations in India was approximately 30%. The prevalence of mortality ranged from 1–21% among ethnic minorities and 4–25% among non-ethnic minorities in HIC and the pooled prevalence of deaths among hospitalizations ranged from 8–15% in India. These estimates suggest that the burden of H1N1 was significant across LIC/LMIC and HIC.

Our results are similar to previous reviews on the global burden of H1N1. In a systematic review of H1N1 in the Southern Hemisphere, 17–45% of laboratory-confirmed H1N1 cases were hospitalized, of which 8–26% were admitted to ICUs and 14–22% died [5]. A similar burden was observed in the Northern Hemisphere, with 94% of patients hospitalized, 36% of patients admitted to ICU, and 39% died [6]. The current review is more comprehensive, with a scope encompassing all regions of the world and comparisons drawn across ethnic minorities in HIC and individuals from LIC/LMIC.

Another study pooled data on risk factors for acquiring H1N1 pandemic and included data from governmental surveillance programs across 19 countries [81]. In this study, ethnic minorities had a higher risk of hospitalization and fatality compared to the general population in Canada, Australia, and New Zealand. For severe H1N1 pandemic, ethnic minorities were under-represented among cases in Thailand and Mexico [81]. Meta-analysis results were not reported due to the small number of countries reporting data.

Some of the included studies compared the burden of H1N1 for ethnic minorities versus non-ethnic minorities in HIC, allowing comparisons between these groups. In two large North American studies, there were significantly more hospitalizations among ethnic minorities versus non-ethnic minorities [25], [38]. However, there were no differences in ICU admissions or deaths among patients hospitalized with H1N1 for ethnic minorities and non-ethnic minorities among studies conducted in North America and Australia. It is possible that non-ethnic minorities had a greater proportion of comorbidities, pregnancy or obesity – known risk factors for pandemic H1N1 (i.e., confounding variables) [5], [6]. However, we did not find any differences in these factors between ethnic minorities and non-ethnic minorities in HIC across some of the studies that were included in the meta-analyses.

Pregnancy is a recognized risk factor for seasonal influenza and pandemic influenza [5], [6], [82]. One of the studies included pregnant women infected with H1N1 and the results were inconsistent across the outcomes examined [28]. For example, significantly higher hospitalizations were observed for pregnant ethnic minorities versus pregnant non-ethnic minorities, yet a significantly higher proportion of deaths occurred among pregnant non-ethnic minorities versus pregnant ethnic minorities [28]. These results could be because non-ethnic minorities had a greater proportion of comorbidity, but this information was not reported. Only one study reported this data so we were unable to examine pregnancy further through meta-analysis.

A small number of seniors were infected with the pandemic A/H1N1/2009 virus, which is inconsistent with previous influenza pandemics [5]. This is likely because seniors were previously exposed to a similar H1N1 strain [83], affording some protection against the 2009 pandemic. However, the elderly still experienced high hospitalization and death rates [5], [6]. We were unable to examine this as none of the included studies reported data specific to elderly socially disadvantaged individuals.

There are limitations associated with the conduct of our systematic review. First, we were unable to include some studies if they did not provide a breakdown for the outcomes of interest by ethnicity. Furthermore, we found that because the classification of ethnicity varies by region, drawing comparisons across countries was difficult [84]. Second, some potentially relevant studies were excluded if they did not isolate confirmed cases from suspected cases in their analysis. Third, some of the included studies did not report details, such as total number of individuals with H1N1, record of potential confounding variables or number lost to follow-up. For these reasons, the systematic review conduct was challenging.

It is possible that relevant unpublished studies were omitted, although efforts were made to contact authors and request access to data that had been presented at conferences. Through these efforts, we successfully obtained the results from two unpublished studies [33], [35]. As well, it’s worth noting that due to the small number of studies included in the meta-analysis of HIC, we were unable to assess the impact of publication bias on our results (i.e., through the use of a funnel plot). Finally, our results are generalizable only to ethnic-minorities in HIC and individuals in LIC/LMIC, as none of the identified studies reported data for other types of social disadvantage (e.g., groups without access or disproportionate access to healthcare or individuals of low socioeconomic status).

To conclude, the prevalence of hospitalization, severe illness, and mortality due to H1N1 was high for ethnic minorities in HIC and individuals from LIC/LMIC. In addition, there was an increased proportion of hospitalization among ethnic minorities compared to non-ethnic minorities in two studies conducted in North America. However, a similar risk of ICU admission and death was observed among ethnic minorities and non-ethnic minorities in studies conducted in Canada, the United States, and Australia. These results suggest that there was little difference in H1N1 burden between ethnic minorities and non-ethnic minorities living in HIC.

## Supporting Information

Appendix S1 Medline search strategy.
(DOCX)

Appendix S2 Methodological quality.
(DOCX)

Appendix S3 Confounding variables and meta-analysis results.
(DOCX)

Flowchart S1 (DOCX)

Checklist S1 (DOCX)
