# Supplementary material for: Impact of H1N1 on Socially Disadvantaged Populations: Systematic Review
Source: PLoS One. 2012 Jun 25;7(6):e39437. doi: 10.1371/journal.pone.0039437 (PMC3382581; doi:10.1371/journal.pone.0039437)
Supplement: Appendix S1 — Medline search strategy. (DOCX) [file pone.0039437.s001.docx]

# APPENDIX S1: MEDLINE SEARCH STRATEGY

Database: Ovid MEDLINE(R) <1948 to July Week 2 2011>, Ovid MEDLINE(R) In-Process & Other Non-Indexed Citations <July 22, 2011>

Search Strategy:

--------------------------------------------------------------------------------

1 H1N1.mp.

2 (swine adj2 (influenza or flu)).tw.

3 exp Influenzavirus A/

4 (influenza adj A).mp.

5 or/1-4

6 exp American Native Continental Ancestry Group/

7 Ethnic Groups/

8 Health Services, Indigenous/

9 exp Homeless Persons/

10 Medical Indigency/

11 Oceanic Ancestry Group/

12 exp Public Assistance/

13 exp Poverty/

14 Socioeconomic Factors/

15 exp Social Welfare/

16 exp Social Security/

17 "Transients and Migrants"/

18 Vulnerable Populations/

19 aborig$.mp.

20 (american adj indian$).tw.

21 disadvantag$.tw.

22 dispossess$.tw.

23 destitut$.tw.

24 homeless$.tw.

25 impover$.tw.

26 indigen$.mp.

27 itinerant$.tw.

28 inuit$.mp.

29 (low adj income$).mp.

30 maor$.mp.

31 minorit$.mp.

32 (native adj american$).tw.

33 (poverty$ or poor$).mp.

34 remote$.tw.

35 (tribe$ or tribal$).tw.

36 transient$.tw.

37 (underprivileg$ or under-privileg$).tw.

38 (vulnerabl$ adj (communit$ or group$ or individual$ or famil$ or population$ or people$ or person$)).tw.

39 (isolat$ adj (communit$ or group$ or individual$ or famil$ or population$ or people$ or person$)).tw. (3266)

40 or/6-39

41 5 and 40

42 Animals/ not (Animals/ and Humans/)

43 41 not 42

44 limit 43 to yr="2009 - 2011"
