# Supplementary material for: Impact of H1N1 on Socially Disadvantaged Populations: Systematic Review
Source: PLoS One. 2012 Jun 25;7(6):e39437. doi: 10.1371/journal.pone.0039437 (PMC3382581; doi:10.1371/journal.pone.0039437)
Supplement: Appendix S2 — Methodological quality. (DOCX) [file pone.0039437.s002.docx]

# APPENDIX S2: METHODOLOGICAL QUALITY

Newcastle Ottawa Scale for cohort studies

**Selection**

1) Representativeness of the exposed cohort

a) truly representative of the average individual infected with H1N1 in the community

b) somewhat representative of the average individual infected with H1N1 in the community

c) selected group of users (e.g., nurses, volunteers)

d) no description of the derivation of the cohort

2) Selection of the non exposed cohort

a) drawn from the same community as the exposed cohort

b) drawn from a different source

c) no description of the derivation of the non exposed cohort

3) Ascertainment of exposure

a) secure record (e.g., surgical records)

b) structured interview

c) written self report

d) no description

4) Demonstration that outcome of interest was not present at start of study

a) yes

b) no

**Comparability**

1) Comparability of cohorts on the basis of the design or analysis

a) study controls for age or gender

b) study controls for any additional factor (e.g., body mass index, comorbidity)

c) no control

**Outcome**

1) Assessment of outcome

a) independent blind assessment

b) record linkage

c) self report

d) no description

2) Was follow-up long enough for outcomes to occur

a) yes (at least 1 week follow-up)

b) no

3) Adequacy of follow up of cohorts

a) complete follow up - all subjects accounted for

b) subjects lost to follow up unlikely to introduce bias - small number lost (<10%)

c) loss to follow-up >10%

d) no statement
