# Supplementary material for: Impact of H1N1 on Socially Disadvantaged Populations: Systematic Review
Source: PLoS One. 2012 Jun 25;7(6):e39437. doi: 10.1371/journal.pone.0039437 (PMC3382581; doi:10.1371/journal.pone.0039437)
Supplement: Appendix S3 — Confounding variables and meta-analysis results. (DOCX) [file pone.0039437.s003.docx]

# APPENDIX S3: CONFOUNDING VARIABLES AND META-ANALYSIS RESULTS

| **Confounding variable** | **Number of studies included in the confounding meta-analyses** | **Number of studies overlapping with the main meta-analyses** | **Number of studies included in the main meta-analyses** |
| --- | --- | --- | --- |
| Co-morbidities | 6 studies [20,24,29,30,43,49];(N=1,203 patients, OR 1.14, 95% CI: 0.63-2.06) | 1[30]    2 [24,30] | 8 studies[25,26,30,35,44,51,59,62] (pooled OR, ICU admissions: 0.84, 95% CI: 0.69-1.02)  6 studies [24,26,30,35,44,51]; (pooled OR, deaths: 0.85, 95% CI: 0.73-1.01) |
| Pregnancy | 4 studies [30,43,46,49]; (N=765 patients, OR 0.31, 95% CI: 0.03-3.64) | 1 [30] | 8 studies [25,26,30,35,44,51,59,62]; (pooled OR, ICU admissions: 0.84, 95% CI: 0.69-1.02) |
| Obesity | 3 studies [20,30,49]; (N=500 patients, OR 0.76, 95% CI: 0.46-1.26) | 1 [30] | 8 studies [25,26,30,35,44,51,59,62]; (pooled OR, ICU admissions: 0.84, 95% CI: 0.69-1.02) |
