# Supplementary material for: Impact of H1N1 on Socially Disadvantaged Populations: Systematic Review
Source: PLoS One. 2012 Jun 25;7(6):e39437. doi: 10.1371/journal.pone.0039437 (PMC3382581; doi:10.1371/journal.pone.0039437)
Supplement: Flowchart S1 — (DOCX) [file pone.0039437.s004.docx]

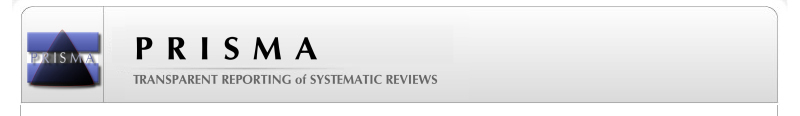
**PRISMA 2009 Flow Diagram S1**

Full-text articles excluded, (n = 102)

1. Not socially disadvantaged (n=70)

2. No relevant outcomes (n=11)

3. Not inflected with A/H1N1/2009 (n=11)

4. No original data (n=9)

5.Not lab confirmed influenza (n=1)

Records excluded (n =623)

1. No relevant outcomes (n=324)

2. Not infected with A/H1N1/2009 (n=101)

3. No original data (78)

4. Not humans (n=49)

5. Case reports (n=41)

6. Not socially disadvantaged (n=20)

7. Conference proceedings (n=10)

Records screened
(n =787)

Full-text articles assessed for eligibility
(n =164)

Studies included in quantitative synthesis (meta-analysis)
(n =48)

*14 companion reports

Studies included in qualitative synthesis
(n =62)

Records after duplicates removed
(n =787)

Additional records identified through other sources
(n =105)

## Identification

## Eligibility

## Included

## Screening

Records identified through database searching
(n = 896)
